# Supplementary material for: Model Checking to Assess T-Helper Cell Plasticity
Source: Front Bioeng Biotechnol. 2015 Jan 28;2:86. doi: 10.3389/fbioe.2014.00086 (PMC4309205; doi:10.3389/fbioe.2014.00086)
Supplement: Supplementary file 1 [file Presentation_1.PDF]

Frontiers in Bioengineering and Biotechnology

Research Article

Supplementary Material: Model checking to assess T-helper cell plasticity

Wassim Abou-Jaoudé [1,2,3,4,†], Pedro T. Monteiro [5,6,†], Aurélien Naldi [7], Maximilien Grandclaudon [8,9], Vassili Soumelis [8,9], Claudine Chaouiya [6] and Denis Thieffry [1,2,3,*]

[1] *Institut de Biologie de l'Ecole Normale Supérieure, Paris, France*
[2] *UMR CNRS 8197, 75005 Paris, France*
[3] *INSERM U1024, 75005 Paris, France*
[4] *Laboratoire d'Informatique de l'Ecole Normale Supérieure, Paris, France*
[5] *INESC-ID, Lisboa, Portugal*
[6] *Instituto Gulbenkian de Ciência, Oeiras, Portugal*
[7] *Centre Intégratif de Génomique, Université de Lausanne, Switzerland*
[8] *Laboratoire d'Immunologie Clinique - Institut Curie, Paris, France*
[9] *INSERM U932, Paris, France*

Correspondence*:
Denis Thieffry
Institut de Biologie de l'Ecole Normale Supérieure (IBENS), 46 Rue d'Ulm, 75005 Paris, France, thieffry@ens.fr

1 SUPPLEMENTARY TABLES AND FIGURES

Supplementary Table S1a. List of the components considered in the Th logical model, with their regulators and supporting evidence. Cell types are CD4 T cells except if another cell type is mentionned. Binding data correspond to experimental evidence on either protein-protein or protein-gene interaction between a component and its regulator. The regulatory graph of the model (shown in Figure 2 of the core text) was built on the basis of these molecular data.

**Component(s)**	**Description**	**Regulated by (interaction sign)**	**Support**		
			**Reference**	**Species/Cell type**	**Comments**
**IL{1A,1B}_e, TGFB_e, IFN{A,B,G}_e, IL{2,4,6,10,12,15}_e, IL{18,21,23,25,27}_e, IL{29,33,36}_e**	External cytokines				
**APC**	Antigen Presenting Cells				
**IFNAR{1,2}, IFNGR{1,2}, IL{4,6,10,15,18}RA, IL{27,28}RA, IL1RL2, IL{2,10,17}RB, IL{1,18}RAP, IL12RB1, GP130, CGC, ST2**	Subchains of the cytokine receptors				
**IL12RB2**	Subchain of the IL-12 receptor	STAT6 (-)	Assumed to reproduce the inhibition of IL12RB2 expression by IL4 (**Szabo et al.** (1997), Fig 1)		
**IL1R1**	Subchain of IL-1 receptor	STAT3 (+)	Assumed to account for ILR1 differential expression in naive Th and Th17 cells (**Vigne et al.** (2012), Fig 1B)		
**IL2RA**	Subchain of the IL-2 receptor	SMAD3 (+)	**Kim et al.** (2006)	Mice	Functional data
		FOXP3 (+)		T cells	Functional data
		NFKB (+)		T cells	Binding and functional data
		NFAT (+)		Mice, T cells	Binding and functional data
		STAT5 (+)		T cells	Binding and functional data
**IL{1,2,4,6,10,12,15,18}R, IL{21,23,25,27}R, IL{29,33,36}R, IFN{A,G}R, TGFBR**	Cytokine receptors	Activated by its subchain(s) and by its associated cytokine(s) (external or secreted)			
**IL23R**	IL-23 receptor	Activated by its subchains and its associated cytokine			
		RORGt (+)	**Zhou et al.** (2007) (Fig 1B)	Mice	Functional data
		STAT3 (+)	**Zhou et al.** (2007) (Fig 5C)	Mice	Functional data
**IL18R**	IL-18 receptor	Activated by its subchains and its associated cytokine			
		STAT4 (+)	**Nakahira et al.** (2001) (Fig 4)	Mice	Functional data
			**Yu et al.** (2007) (Fig 5C)		Binding data
**TCR, CD28**	T Cell Receptor and its co-receptor	APC (+)	**Chen and Flies** (2013)		
**proliferation**	Cell proliferation	STAT5 (+)	**Moriggl et al.** (1999) (Fig 3)	T cells, Mice	Functional data
		proliferation (+)	Assumed		
**IKB**	IκB	TCR (-)	**Weil and Israel** (2006)	T cells	Functional data
**NFKB**	NFκB	FOXP3 (-)	**Bettelli et al.** (2005) (Fig 2, 3 and 4)	Mice	Binding and functional data
		IKB (-)	**Weil and Israel** (2006)	T cells	Binding and functional data
**NFAT**	Transcription factor	TCR, CD28 (+)	**Diehn et al.** (2002)	T cells, Human	Functional data
**STAT1**	Transcription factor	IL27R (+)	**Kamiya et al.** (2004) (Fig 1)	Mice	Functional data
		IFNAR, IFNGR (+)	**Kotenko and Pestka** (2000); **Horvath et al.** (1995) (Fig 4)	Human	Functional data
**STAT3**	Transcription factor	IL10R (+)	**Finbloom and Winestock** (1995) (Fig 5)	T cells, Human	Functional data
		IL23R (+)	**Parham et al.** (2002) (Fig 6A)	Human	Functional data
		IL6R (+)	**Croker et al.** (2003) (Fig 3D);	Mice	Functional data
		IL27R (+)	**Charlot-Rabiega et al.** (2011) (Fig 4A)	Human	Functional data
		IL21R (+)	**Brenne et al.** (2002) (Fig 7)	Human, Myeloma cells	Functional data
		IL1R (+)	**Maitra et al.** (2009) (Fig 4)	Mice	Functional data
**STAT4**	Transcription factor	IL12R (+)	**Rogge et al.** (1998) (Fig 4A)	Human	Functional data
		GATA3 (-)	**Usui et al.** (2003) (Fig 1)	Mice	Functional data
**STAT5**	Transcription factor	IL2R (+)	**Chen et al.** (2006) (Fig 3C)	Mice	Functional data
		IL15R (+)	**Pandiyan et al.** (2012) (Fig 5)	Mice	Functional data
**STAT6**	Transcription factor	IL4R (+)	**Chen et al.** (2006) (Fig 3B);	Mice	Functional data
**IRF1**	Transcription factor	STAT1 (+)	Assumed to account for IRF1 activation by IFNγ signaling (**Kano et al.** (2008), Fig 2D)		
**RUNX3**	Transcription factor	TBET (+)	**Djuretic et al.** (2007) (Fig 1)	Mice	Functional data
**SMAD3**	Transcription factor	TGFBR (+)	**Yang et al.** (1999)	T cells	Functional data
**cMAF**	Transcription factor	TGFBR (+)	**Xu et al.** (2009) (Fig 1B)	Mice	Functional data
		STAT3 (+)	**Xu et al.** (2009) (Fig 1B)	Mice	Functional data

Supplementary Table S1b. List of the components considered in the Th logical model, with their regulators and supporting evidence. Cell types are CD4 T cells except if another cell type is mentionned. Binding data correspond to experimental evidence on either protein-protein or protein-gene interaction between a component and its regulator.

Component(s)	Description	Regulated by (interaction sign)	Support		
			Reference	Species/Cell type	Comments
**TBET**	Transcription factor<br>Master regulator of Th1	TBET (+)	**Kanhere et al.** (2012) (Fig 5)	Human	Binding and functional data
		STAT1 (+)	**Afkarian et al.** (2002) (Fig 2)	Mice	Functional data
		RORGt (-)	**Mukasa et al.** (2010) (Fig S5B)	Mice	Functional data
		BCL6 (-)	**Yu et al.** (2009) (Fig 4)	Mice	Binding and functional data
		IL36R (+)	**Vigne et al.** (2012) (Fig 2C)	Mice	Functional data
**GATA3**	Transcription factor<br>Master regulator of Th2	STAT6 (+)	**Ouyang et al.** (1998) (Fig 1C)	Mice	Functional data
		GATA3 (+)	**Ouyang et al.** (2000) (Fig 6A)	Mice	Functional data
		TBET (-)	**Usui et al.** (2006) (Fig 5)<br>**Hwang et al.** (2005b) (Fig 3)	Mice	Binding and functional data
		IL25R (+)	**Wang et al.** (2007) (Fig 4)	Human	Functional data
		IL29R (-)	**Dai et al.** (2009) (Fig 5)	Human	Functional data
		BCL6 (-)	**Nurieva et al.** (2009) (Fig S2C)	Mice	Functional data
		PU1 (-)	**Chang et al.** (2005) (Fig 6,7)	Mice	Binding and functional data
**RORGt**	Transcription factor<br>Master regulator of Th17	STAT3 (+)	**Laurence et al.** (2007) (Fig 4C)	Mice	Functional data
		TGFBR (+)	**Ivanov et al.** (2006) (Fig 3C)	Mice	Functional data
		BCL6 (-)	**Yu et al.** (2009) (Fig 4A)		Binding data
			**Nurieva et al.** (2009) (Fig 3C)	Mice	Functional data
		FOXP3 (-)	**Zhou et al.** (2008) (Fig 3)	Mice	Binding and functional data
**FOXP3**	Transcription factor<br>Master regulator of Treg	STAT5 (+)	**Yao et al.** (2007) (Fig 6A and 7)	Mice	Binding and functional data
			**Jenks et al.** (2013) (Fig 2D)	Human	Functional data
		FOXP3 (+)	**Zheng et al.** (2010) (Fig 4)	Mice	Binding and functional data
		NFAT, SMAD3 (+)	**Tone et al.** (2008) (Fig 4, 6 and 7)	Mice	Binding and functional data
		STAT3 (-)	**Laurence et al.** (2012) (Fig 5A)	Mice	Functional data
		STAT1 (-)	Assumed from putative binding data (**Floess et al.** (2007) Fig S1)		
		RORGt (-)	**Burgler et al.** (2010) (Fig 1, 2, 4 and 5)	Human	Binding and functional data
		STAT6 (-)	**Dardalhon et al.** (2008) (Fig 2B and 2C)	Mice	Functional data
**BCL6**	Transcription factor<br>Master regulator of Tfh	STAT1 (+)	**Choi et al.** (2013) (Fig 3 and 4)	Mice	Functional data
		STAT3 (+)	**Choi et al.** (2013) (Fig 2 and 3)	Mice	Functional data
		STAT5 (-)	**Johnston et al.** (2012) (Fig 2)	Mice	Functional data
		TBET (-)	**Nakayamada et al.** (2011) (Fig 4D)	Mice	Functional data
		STAT4 (+)	**Nakayamada et al.** (2011) (Fig 2E)	Mice	Functional data
**PU1**	Transcription factor specific to Th9	TGFBR (+)	**Chang et al.** (2010) (Fig 1D)	Mice	Functional data
**IFNG**	IFNγ	TBET (+)	**Djuretic et al.** (2007) (Fig 2 and 3)	Mice	Binding and functional data
		RUNX3 (+)	**Djuretic et al.** (2007) (Fig 2 and 3)	Mice	Binding and functional data
		STAT4 (+)	**Afkarian et al.** (2002) (Fig 3A)	Mice	Functional data
			**Balasubramani et al.** (2010) (Fig 4B and S4)		Binding data
		NFAT (+)	**Kiani et al.** (2001) (Fig 1)	Mice	Functional data
		proliferation (+)	Assumed		
		FOXP3 (-)	**Bettelli et al.** (2005) (Fig 1)	Mice	Functional data
		IL18R (+)	**Blom and Poulsen** (2012) (Fig 2A)	Human	Functional data
**TGFB**	TGF-β	FOXP3 (+)	Assumed to account for TGFb production by Treg cells (**Vignali et al.** (2008))		
		NFAT, proliferation (+)	Assumed		
**IL2**	Interleukin-2	NFKB (+)	**Hwang et al.** (2005a) (Fig 6 and 7)	Mice	Binding and functional data
		NFAT (+)	**Peng et al.** (2001) (Fig 2C)	Mice	Functional data
		FOXP3 (-)	**Bettelli et al.** (2005) (Fig 1A)	Mice	Functional data
		TBET (-)	**Hwang et al.** (2005a) (Fig 6)	Mice	Binding and functional data
		STAT5 (-)	**Villarino et al.** (2007) (Fig 3)	Mice	Functional data
		STAT6 (-)	**Villarino et al.** (2007) (Fig 4)	Mice	Functional data
**IL3**	Interleukin-3	GATA3 (+)	**Kitamura et al.** (2005) (Fig 3)	Jurkat cells	Functional data
		NFAT, proliferation (+)	Assumed		
**IL4**	Interleukin-4	GATA3 (+)	**Ouyang et al.** (2000) (Fig 5A)	Mice	Functional data
			**Agarwal et al.** (2000) (Fig 7)		Binding data
		NFAT (+)	**Bettelli et al.** (2005) (Fig 3A)	Mice	Functional data
		proliferation (+)	Assumed		
		FOXP3 (-)	**Bettelli et al.** (2005) (Fig 1B)	Mice	Functional data
		TBET, RUNX3 (-)	**Djuretic et al.** (2007) (Fig 5A and 7)	Mice	Binding and functional data
		IRF1 (-)	**Elser et al.** (2002) (Fig 3 and Table 1)	Mice	Binding and functional data
		STAT5 (+)	**Zhu et al.** (2003) (Fig 1 and 7)	Mice	Functional and binding data
		cMAF (+)	**Nurieva et al.** (2003) (Fig 3C)	Mice	Functional data
**IL5**	Interleukin-5	GATA3 (+)	**Zhu et al.** (2004) (Fig 3A)<br>**Zhang et al.** (1998) (Fig 4B)	Mice	Binding and functional data
		IL33R (+)	**Kurowska-Stolarska et al.** (2008) (Fig 1C)	Human	Functional data
		cMAF (+)	**Nurieva et al.** (2003) (Fig 3C)	Mice	Functional data
		NFAT, proliferation (+)	Assumed		
		FOXP3 (-)	**Dardalhon et al.** (2008) (Fig 3D)	Mice	Functional data
**IL6**	Interleukin-6	STAT3 (+)	Assumed to reproduce IL6 production in proTh17 condition (**Volpe et al.** (2008))		
		NFAT, proliferation (+)	Assumed		
**IL9**	Interleukin-9	NFKB (+)	**Jash et al.** (2012) (Fig 5, 6 and 7)	Mice	Functional and binding data
		IL33R (+)	**Blom et al.** (2011) (Fig 5)	Human	Functional data
		STAT6 (+)	**Veldhoen et al.** (2008) (Fig 2A and 2C)	Mice	Functional data
		NFAT (+)	**Jash et al.** (2012) (Fig 2 and 3)	Mice	Functional and binding data
		SMAD3 (+)	**Elyaman et al.** (2012) (Fig 4)	Mice	Functional and binding data
		proliferation (+)	Assumed		
		PU1 (+)	**Chang et al.** (2010) (Fig 4 and 5)	Mice	Binding and functional data

Supplementary Table S1c. List of the components considered in the Th logical model, with their regulators and supporting evidence. Cell types are CD4 T cells except if another cell type is mentionned. Binding data correspond to experimental evidence on either protein-protein or protein-gene interaction between a component and its regulator.

Component(s)	Description	Regulated by (interaction sign)	Support		
			Reference	Species/Cell type	Comments
**IL10**	Interleukin-10	GATA3 (+)	**Shoemaker et al.** (2006) (Fig 2 and 4)	Mice	Functional data
		STAT3 (+)	**Ziegler-Heitbrock et al.** (2003) (Fig 6)	Human	Functional data
		NFAT, proliferation (+)	Assumed		
		STAT4 (+)	**Saraiva et al.** (2009) (Fig 2A and 3)	Mice	Functional data
		IRF1 (+)	**Ziegler-Heitbrock et al.** (2003) (Fig 1)	Human	Binding data
		cMAF (+)	**Xu et al.** (2009) (Fig 5 and 6)	Mice	Functional and binding data
		IL18R (-)	**Blom and Poulsen** (2012) (Fig 2A)	Human	Functional data
		IL33R (-)	**Blom and Poulsen** (2012) (Fig 2A)	Human	Functional data
**IL13**	Interleukin-13	GATA3 (+)	**Zhu et al.** (2004) (Fig 3)	Mice	Functional and binding data
			**Lavenu-Bombled et al.** (2002) (Fig 6 and 7)		
		cMAF (+)	**Nurieva et al.** (2003) (Fig 3C)	Mice	Functional data
		IL33R (+)	**Kurowska-Stolarska et al.** (2008) (Fig 1C)	Human	Functional data
		NFAT, proliferation (+)	Assumed		
		FOXP3 (-)	**Dardalhon et al.** (2008) (Fig 3C)	Mice	Functional data
**IL17**	Interleukin-17	RORGt (+)	**Ivanov et al.** (2006) (Fig 3A)	Mice	Functional data
		STAT3 (+)	**Chen et al.** (2006) (Fig 3E)	Mice	Binding and functional data
			**Laurence et al.** (2007) (Fig 4A)		
		NFAT, proliferation (+)	Assumed		
		NFKB (+)	Assumed		
		STAT5 (-)	**Laurence et al.** (2007) (Fig 5 and 6)	Mice	Binding and functional data
		FOXP3 (-)	**Zhou et al.** (2008) (Fig 3C)	Mice	Functional data
		STAT1 (-)	**Laurence et al.** (2007)	Mice	Binding data
		STAT6 (-)	**Laurence et al.** (2007)	Mice	Binding data
**IL21**	Interleukin-21	NFAT, proliferation (+)	Assumed		
		STAT3 (+)	**Durant et al.** (2010) (Fig 4)	Mice	Functional and binding data
			**Zhou et al.** (2007) (Fig 5B)		
		STAT4 (+)	**Schmitt et al.** (2009) (Fig 6)	Human	Functional data
		cMAF (+)	**Bauquet et al.** (2009) (Fig 6E)	Mice	Functional data
**IL22**	Interleukin-22	STAT3 (+)	**Yang et al.** (2007) (Fig 2D and 3C)	Mice	Functional data
		cMAF (-)	**Rutz et al.** (2011) (Fig 3, 4, 7 and 8)	Mice	Functional and binding data
		NFAT, proliferation (+)	Assumed		
		STAT1 (+)	Assumed to reproduce IL22 production in IL12 condition (**Volpe et al.** (2008), Fig 5A)		
**IL24**	Interleukin-24	STAT6 (+)	**Sahoo et al.** (2011) (Fig 5, 6 and 8)	Mice	Functional and binding data
		proliferation, NFAT (+)	Assumed		
**IL25**	Interleukin-25	GATA3 (+)	Assumed to reproduce IL25 production in Th2 cells (**Paul and Zhu** (2010))		
		proliferation, NFAT (+)	Assumed		
**IL31**	Interleukin-31	STAT6 (+)	**Park et al.** (2012) (Fig 5, 7 and 8)	Human, Jurkat cells	Functional and binding data
		NFAT (+)	**Park et al.** (2012) (Fig 5 and 6)	Human, Jurkat cells	Functional and binding data
		proliferation (+)	Assumed		
**IL35**	Interleukin-35	FOXP3 (+)	**Collison et al.** (2007) (Fig S5B)	Mice	Functional data
		proliferation, NFAT (+)	Assumed		

Supplementary Table S2a. Logical functions associated with the membrane receptor components of the model. Operators "∧", "∨" and "¬" stand for AND, OR and NOT, respectively. Note that the input components are omitted. These input components (all Boolean) represent cytokine environments and APC: IL1B_e, IFNG_e, IL2_e, IL4_e, IL6_e, IL10_e, IL12_e, IL15_e, IL21_e, IL23_e, IL27_e, TGFB_e, IL36_e, IL33_e, IL18_e, IL25_e, IFNB_e, IFNA_e, IL1A_e, IL29_e and APC. Their values are defined in the initial conditions and maintained throughout simulations.

	Component	Target value	Logical function
**Membrane receptors**	**TCR**	1	APC
	**CD28**	1	APC
	**IFNGR**	1	IFNGR1 ∧ IFNGR2 ∧ (IFNG ∨ IFNG_e)
	**IFNGR1**	1	(basal value)
	**IFNGR2**	1	(basal value)
	**IL36R**	1	IL36_e ∧ IL1RL2 ∧ IL1RAP
	**IL1RL2**	1	(basal value)
	**IL1R**	1	(IL1B_e ∨ IL1A_e) ∧ IL1RAP ∧ IL1R1
	**IL1R1**	1	STAT3
	**IL1RAP**	1	(basal value)
	**IL2R**	1	CGC ∧ IL2RB ∧ ¬IL2RA ∧ (IL2 ∨ IL2_e)
		2	CGC ∧ IL2RB ∧ IL2RA ∧ (IL2 ∨ IL2_e)
	**IL2RB**	1	(basal value)
	**IL2RA**	1	(SMAD3 ∨ FOXP3 ∨ STAT5 ∨ NFKB) ∧ NFAT
	**IL4R**	1	CGC ∧ IL4RA ∧ (IL4 ∨ IL4_e)
	**IL6R**	1	GP130 ∧ IL6RA ∧ (IL6_e ∨ IL6)
	**IL10R**	1	IL10RA ∧ IL10RB ∧ (IL10 ∨ IL10_e)
	**IL12R**	1	IL12RB1 ∧ IL12RB2 ∧ IL12_e
	**IL15R**	1	CGC ∧ IL15RA ∧ IL2RB ∧ IL15_e
	**IL21R**	1	GP130 ∧ CGC ∧ (IL21 ∨ IL21_e)
	**IL23R**	1	GP130 ∧ IL12RB1 ∧ IL23_e ∧ STAT3 ∧ RORGT
	**IL27R**	1	GP130 ∧ IL27RA ∧ IL27_e
	**IL27RA**	1	(basal value)
	**IFNAR**	1	(IFNA_e ∨ IFNB_e) ∧ IFNAR1 ∧ IFNAR2
	**IFNAR1**	1	(basal value)
	**IFNAR2**	1	(basal value)
	**TGFBR**	1	TGFB ∨ TGFB_e
	**GP130**	1	(basal value)
	**IL6RA**	1	(basal value)
	**IL12RB1**	1	(basal value)
	**IL12RB2**	1	¬STAT6
	**CGC**	1	(basal value)
	**IL10RA**	1	(basal value)
	**IL10RB**	1	(basal value)
	**IL4RA**	1	(basal value)
	**IL15RA**	1	(basal value)
	**IL29R**	1	IL29_e ∧ IL28RA ∧ IL10RB
	**IL17RB**	1	(basal value)
	**IL18RAP**	1	(basal value)
	**IL18RA**	1	(basal value)
	**IL18R**	1	IL18_e ∧ IL18RAP ∧ IL18RA ∧ STAT4
	**ST2**	1	GATA3
	**IL25R**	1	IL17RB ∧ (IL25_e ∨ IL25)
	**IL33R**	1	IL33_e ∧ ST2 ∧ IL1RAP
	**IL28RA**	1	(basal value)

Supplementary Table S2b. Logical functions associated with the intracellular signaling components and the components denoting the secreted cytokines and cell proliferation. Operators "∧", "∨" and "¬" stand for AND, OR and NOT, respectively.

	Component	Target value	Logical function
**Intracellular signaling components**	**IKB**	1	¬TCR
	**NFKB**	1	¬IKB ∧ ¬FOXP3
	**NFAT**	1	TCR ∧ CD28
	**TBET**	1	(TBET ∨ STAT1 ∨ IL36R) ∧ ¬BCL6 ∧ ¬RORGT
	**GATA3**	1	(¬GATA3 ∧ ¬TBET ∧ (STAT6 ∨ IL25R) ∧ ¬BCL6 ∧ ¬PU1 ∧ ¬IL29R) ∨ (GATA3 ∧ ¬BCL6 ∧ ¬PU1 ∧ ¬IL29R)
	**RORGT**	1	TGFBR ∧ STAT3 ∧ ¬BCL6 ∧ ¬FOXP3
	**FOXP3**	1	(STAT5 ∧ NFAT ∧ FOXP3 ∧ ¬STAT6) ∨ (STAT5 ∧ NFAT ∧ ¬FOXP3 ∧ SMAD3 ∧ ¬STAT1 ∧ ¬(STAT3 ∧ RORGT) ∧ ¬STAT6)
	**BCL6**	1	((STAT1 ∨ STAT3 ∨ STAT4) ∧ ¬TBET ∧ ¬STAT5) ∨ (STAT3 ∧ STAT4 ∧ ¬TBET)
	**STAT1**	1	IFNAR ∨ IFNGR ∨ IL27R
	**STAT3**	1	IL6R ∨ IL23R ∨ IL1R ∨ IL21R ∨ IL27R
	**STAT4**	1	IL12R ∧ ¬GATA3
	**STAT5**	1	¬IL2R:2 ∧ (IL2R:1 ∨ IL15R)
		2	IL2R:2
	**STAT6**	1	IL4R
	**cMAF**	1	TGFBR ∧ STAT3
	**PU1**	1	TGFBR
	**SMAD3**	1	TGFBR
	**IRF1**	1	STAT1
	**RUNX3**	1	TBET
**Secreted cytokines**	**IFNG**	1	proliferation ∧ ¬FOXP3 ∧ NFAT ∧ ((TBET ∧ RUNX3) ∨ STAT4 ∨ IL18R)
	**IL4**	1	NFAT ∧ proliferation ∧ GATA3 ∧ (STAT5 ∨ cMAF) ∧ ¬FOXP3 ∧ ¬((TBET ∧ RUNX3) ∨ IRF1)
	**IL2**	1	(NFAT ∨ NFKB) ∧ ¬TBET ∧ ¬FOXP3 ∧ ¬(STAT5 ∧ STAT6)
	**IL17**	1	NFAT ∧ proliferation ∧ RORGT ∧ NFKB ∧ STAT3 ∧ ¬(FOXP3 ∧ STAT1 ∧ STAT5 ∧ STAT6)
	**IL22**	1	proliferation ∧ NFAT ∧ (STAT3 ∨ STAT1) ∧ ¬cMAF
	**IL9**	1	(NFKB ∨ NFAT) ∧ proliferation ∧ (SMAD3 ∨ PU1 ∨ IL33R) ∧ STAT6
	**IL10**	1	(GATA3 ∨ STAT3 ∨ STAT4 ∨ cMAF ∨ IRF1) ∧ NFAT ∧ proliferation ∧ ¬IL18R ∧ ¬IL33R
	**IL3**	1	GATA3 ∧ proliferation ∧ NFAT
	**IL21**	1	NFAT ∧ proliferation ∧ (STAT3 ∨ cMAF ∨ STAT4)
	**IL5**	1	proliferation ∧ NFAT ∧ (GATA3 ∨ cMAF ∨ IL33R) ∧ ¬FOXP3
	**IL13**	1	proliferation ∧ NFAT ∧ (GATA3 ∨ cMAF ∨ IL33R) ∧ ¬FOXP3
	**IL6**	1	proliferation ∧ NFAT ∧ STAT3
	**TGFB**	1	NFAT ∧ proliferation ∧ FOXP3
	**IL35**	1	NFAT ∧ proliferation ∧ FOXP3
	**IL25**	1	NFAT ∧ proliferation ∧ GATA3
	**IL31**	1	NFAT ∧ proliferation ∧ STAT6
	**IL24**	1	NFAT ∧ proliferation ∧ STAT6
	**proliferation**	1	STAT5:2 ∨ proliferation

Supplementary Table S3. Context-dependent stable states identified by GINsim. A red (resp. green) cell denotes the activation (resp. inactivation) of the corresponding component (column entries) for the corresponding stable state pattern (row entries). Gray cells indicate components that are either activated or inactivated. Note that the values of the input nodes are omitted. A state stable for a given input combination may become unstable for other input values. Stable states are associated with Th cell phenotypes (row index) according to the expression of specific markers. *Inactive* Th cells denote cells that do not express their characteristic cytokines.

	Transcription factors							Cytokines																		
	TBET	GATA3	RORγT	FOXP3	BCL6	PU.1	STAT3	IFNγ	IL4	IL17	IL5	IL13	TGFβ	IL9	IL22	IL21	IL2	IL10	IL6	IL3	IL24	IL25	IL31	IL35	prolif	82 states
**Th0**																										2
**Activated Th0**																										1
**Th1**																										7
**Inactive Th1**																										6
**Th2**																										4
**Inactive Th2**																										4
**Th17**																										2
**Inactive Th17**																										2
**Treg**																										2
**Inactive Treg**																										4
**Tfh**																										4
**Inactive Tfh**																										8
**Th9**																										1
**Inactive Th9**																										2
**Th22**																										2
**Inactive Th22**																										2
**Hybrid Th Gata3⁺Foxp3⁺**																										2
**Hybrid Th Tbet⁺Foxp3⁺**																										8
**Hybrid Th Tbet⁺Gata3⁺**																										12
**Hybrid Th Foxp3⁺Bcl6⁺**																										4
**Hybrid Th Tbet⁺Gata3⁺Foxp3⁺**																										2
**IL24⁺IL31⁺**																										1

Supplementary Table S4. Model checking of the polarization from naive Th cells (Th0) to the triple hybrid Th subtype (Tbet+Gata3+Foxp3+) predicted by the model in a selection of input cytokine conditions. Each row corresponds to an ARCTL formula specification, having the generic property: INIT Th0; $\mathsf{EAF}(e_1)(\mathtt{true} \wedge \mathsf{EAF}(e_2)(\text{Tbet}^+\text{Gata3}^+\text{Foxp3}^+ \wedge \mathsf{AAG}(e_2)(\text{Tbet}^+\text{Gata3}^+\text{Foxp3}^+)))$, where e_1 and e_2 correspond to input condition 1 and input condition 2 respectively, and Tbet+Gata3+Foxp3+ is the hybrid Th stable pattern defined in Supplementary Table S3. Each of these input conditions has a fixed input valuation for the variables specified in the table, letting all other input variables freely vary (+/- denotes the presence/absence of cytokine).

**Input condition 1**			**Input condition 2**		**Model checking**
+ TGFβ + APC			+ IL15 + APC		
IL25	IFNγ	IL15	IL25	IFNγ	
+	+	-	-	+	false
+	+	+	-	+	false
+	+	-	+	+	false
+	+	+	+	+	false
+	-	-	+	+	true
+	-	+	+	+	true
-	+	-	+	+	false
-	+	+	+	+	false
+	+	+	+	-	false
+	+	+	+	-	false
-	-	-	+	+	true
-	-	+	+	+	true
+	+	-	-	-	false
+	+	+	-	-	false
+	-	-	-	+	true
+	-	+	-	+	true
-	+	+	+	-	false
-	+	+	+	-	false

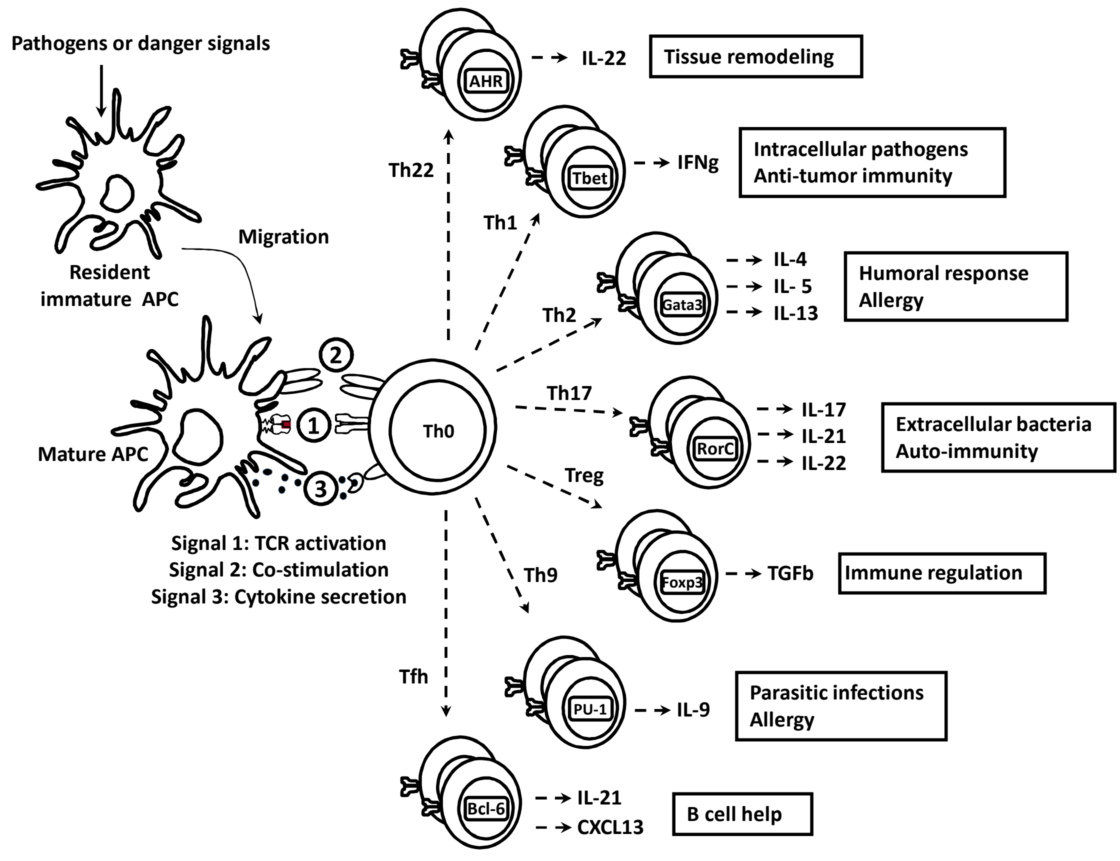

Supplementary Figure S1. The classical model of T helper cell differentiation. After encountering pathogens or danger signals at the periphery, dendritic cell matures and migrates to the secondary lymphoid organs to initiate T helper differentiation. Three types of signals are involved in this process. Classically signals 1 and 2 (TCR activation and co-stimulation) mainly contribute to T cell priming, activation and clonal proliferation. Signal 3 (cytokines) is mainly responsible for the polarization of the naive T cells (Th0) towards distinct lineages, among which the canonical Th subsets Th1, Th2, Th17, Treg, Tfh, Th9 and Th22. These different lineages are characterized by a set of cytokines they express under the control of a 'master regulator' transcriptional factor. Each master regulator is critically involved in the driving of the differentiation of the Th lineage they specify. These subsets are associated to specific physiopathological functions. For instance, Th1 cells express IFNγ as a hallmark cytokine under the control of the master regulator Tbet, which directs Th1 lineage commitment, and is involved in the clearance of intracellular pathogens. Importantly, this classical linear view of Th differentiation into distinct separated subsets has been recently challenged by observations highlighting Th plasticity, including the identification of hybrid Th subsets expressing several master regulators and of reprogramming events between Th subsets under specific cytokine environments.

REFERENCES

Afkarian, M., Sedy, J. R., Yang, J., Jacobson, N. G., Cereb, N., Yang, S. Y., et al. (2002), T-bet is a STAT1-induced regulator of IL-12R expression in nave CD4+ T cells, *Nature Immunology*, 3, 6, 549–557, doi:10.1038/ni794

Agarwal, S., Avni, O., and Rao, A. (2000), Cell-type-restricted binding of the transcription factor NFAT to a distal IL-4 enhancer in vivo, *Immunity*, 12, 6, 643–652, doi:10.1016/S1074-7613(00)80215-0

Balasubramani, A., Shibata, Y., Crawford, G. E., Baldwin, A. S., Hatton, R. D., and Weaver, C. T. (2010), Modular utilization of distal cis-regulatory elements controls Ifng gene expression in T cells activated by distinct stimuli, *Immunity*, 33, 1, 35–47, doi:10.1016/j.immuni.2010.07.004

Bauquet, A. T., Jin, H., Paterson, A. M., Mitsdoerffer, M., Ho, I. C., Sharpe, A. H., et al. (2009), The costimulatory molecule ICOS regulates the expression of c-Maf and IL-21 in the development of follicular T helper cells and TH-17 cells, *Nature Immunology*, 10, 2, 167–175, doi:10.1038/ni.1690

Bettelli, E., Dastrange, M., and Oukka, M. (2005), Foxp3 interacts with nuclear factor of activated T cells and NF-kappa B to repress cytokine gene expression and effector functions of T helper cells, *Proceedings of the National Academy of Sciences U.S.A.*, 102, 14, 5138–5143, doi:10.1073/pnas.0501675102

Blom, L., Poulsen, B. C., Jensen, B. M., Hansen, A., and Poulsen, L. K. (2011), IL-33 induces IL-9 production in human CD4+ T cells and basophils, *PLoS ONE*, 6, 7, e21695, doi:10.1371/journal.pone.0021695

Blom, L. and Poulsen, L. K. (2012), IL-1 family members IL-18 and IL-33 upregulate the inflammatory potential of differentiated human Th1 and Th2 cultures, *Journal of Immunology*, 189, 9, 4331–4337, doi:10.4049/jimmunol.1103685

Brenne, A. T., Ro, T. B., Waage, A., Sundan, A., Borset, M., and Hjorth-Hansen, H. (2002), Interleukin-21 is a growth and survival factor for human myeloma cells, *Blood*, 99, 10, 3756–3762, doi:10.1182/blood.V99.10.3756

Burgler, S., Mantel, P. Y., Bassin, C., Ouaked, N., Akdis, C. A., and Schmidt-Weber, C. B. (2010), RORC2 is involved in T cell polarization through interaction with the FOXP3 promoter, *Journal of Immunology*, 184, 11, 6161–6169, doi:10.4049/jimmunol.0903243

Chang, H. C., Sehra, S., Goswami, R., Yao, W., Yu, Q., Stritesky, G. L., et al. (2010), The transcription factor PU.1 is required for the development of IL-9-producing T cells and allergic inflammation, *Nature Immunology*, 11, 6, 527–534, doi:10.1038/ni.1867

Chang, H. C., Zhang, S., Thieu, V. T., Slee, R. B., Bruns, H. A., Laribee, R. N., et al. (2005), PU.1 expression delineates heterogeneity in primary Th2 cells, *Immunity*, 22, 6, 693–703, doi:10.1016/j.immuni.2005.03.016

Charlot-Rabiega, P., Bardel, E., Dietrich, C., Kastelein, R., and Devergne, O. (2011), Signaling events involved in interleukin 27 (IL-27)-induced proliferation of human naive CD4+ T cells and B cells, *Journal of Biological Chemistry*, 286, 31, 27350–27362, doi:10.1074/jbc.M111.221010

Chen, L. and Flies, D. B. (2013), Molecular mechanisms of T cell co-stimulation and co-inhibition, *Nature Reviews Immunology*, 13, 4, 227–242, doi:10.1038/nri3405

Chen, Z., Laurence, A., Kanno, Y., Pacher-Zavisin, M., Zhu, B. M., Tato, C., et al. (2006), Selective regulatory function of Socs3 in the formation of IL-17-secreting T cells, *Proceedings of the National Academy of Sciences U.S.A.*, 103, 21, 8137–8142, doi:10.1073/pnas.0600666103

Choi, Y. S., Eto, D., Yang, J. A., Lao, C., and Crotty, S. (2013), Cutting edge: STAT1 is required for IL-6-mediated Bcl6 induction for early follicular helper cell differentiation, *Journal of Immunology*, 190, 7, 3049–3053, doi:10.4049/jimmunol.1203032

Collison, L. W., Workman, C. J., Kuo, T. T., Boyd, K., Wang, Y., Vignali, K. M., et al. (2007), The inhibitory cytokine IL-35 contributes to regulatory T-cell function, *Nature*, 450, 7169, 566–569, doi:10.1038/nature06306

Croker, B. A., Krebs, D. L., Zhang, J. G., Wormald, S., Willson, T. A., Stanley, E. G., et al. (2003), SOCS3 negatively regulates IL-6 signaling in vivo, *Nature Immunology*, 4, 6, 540–545, doi:10.1038/ni931

Dai, J., Megjugorac, N. J., Gallagher, G. E., Yu, R. Y., and Gallagher, G. (2009), IFN-lambda1 (IL-29) inhibits GATA3 expression and suppresses Th2 responses in human naive and memory T cells, *Blood*, 113, 23, 5829–5838, doi:10.1182/blood-2008-09-179507

Dardalhon, V., Awasthi, A., Kwon, H., Galileos, G., Gao, W., Sobel, R. A., et al. (2008), IL-4 inhibits TGF-beta-induced Foxp3+ T cells and, together with TGF-beta, generates IL-9+ IL-10+ Foxp3(-) effector T cells, *Nature Immunology*, 9, 12, 1347–1355, doi:10.1038/ni.1677

Diehn, M., Alizadeh, A. A., Rando, O. J., Liu, C. L., Stankunas, K., Botstein, D., et al. (2002), Genomic expression programs and the integration of the CD28 costimulatory signal in T cell activation, *Proceedings of the National Academy of Sciences U.S.A.*, 99, 18, 11796–11801, doi:10.1073/pnas.092284399

Djuretic, I. M., Levanon, D., Negreanu, V., Groner, Y., Rao, A., and Ansel, K. M. (2007), Transcription factors T-bet and Runx3 cooperate to activate Ifng and silence Il4 in T helper type 1 cells, *Nature Immunology*, 8, 2, 145–153, doi:10.1038/ni1424

Durant, L., Watford, W. T., Ramos, H. L., Laurence, A., Vahedi, G., Wei, L., et al. (2010), Diverse targets of the transcription factor STAT3 contribute to T cell pathogenicity and homeostasis, *Immunity*, 32, 5, 605–615, doi:10.1016/j.immuni.2010.05.003

Elser, B., Lohoff, M., Kock, S., Giaisi, M., Kirchhoff, S., Krammer, P. H., et al. (2002), IFN-gamma represses IL-4 expression via IRF-1 and IRF-2, *Immunity*, 17, 6, 703–712, doi:10.1016/S1074-7613(02)00471-5

Elyaman, W., Bassil, R., Bradshaw, E. M., Orent, W., Lahoud, Y., Zhu, B., et al. (2012), Notch receptors and Smad3 signaling cooperate in the induction of interleukin-9-producing T cells, *Immunity*, 36, 4, 623–634, doi:10.1016/j.immuni.2012.01.020

Finbloom, D. S. and Winestock, K. D. (1995), IL-10 induces the tyrosine phosphorylation of tyk2 and Jak1 and the differential assembly of STAT1 alpha and STAT3 complexes in human T cells and monocytes, *Journal of Immunology*, 155, 3, 1079–1090

Floess, S., Freyer, J., Siewert, C., Baron, U., Olek, S., Polansky, J., et al. (2007), Epigenetic control of the foxp3 locus in regulatory T cells, *PLoS Biology*, 5, 2, e38, doi:10.1371/journal.pbio.0050038

Horvath, C. M., Wen, Z., and Darnell, J. E. (1995), A STAT protein domain that determines DNA sequence recognition suggests a novel DNA-binding domain, *Genes and Development*, 9, 8, 984–994, doi:10.1101/gad.9.8.984

Hwang, E. S., Hong, J. H., and Glimcher, L. H. (2005a), IL-2 production in developing Th1 cells is regulated by heterodimerization of RelA and T-bet and requires T-bet serine residue 508, *Journal of Experimental Medecine*, 202, 9, 1289–1300, doi:10.1084/jem.20051044

Hwang, E. S., Szabo, S. J., Schwartzberg, P. L., and Glimcher, L. H. (2005b), T helper cell fate specified by kinase-mediated interaction of T-bet with GATA-3, *Science*, 307, 5708, 430–433, doi:10.1126/science.1103336

Ivanov, I. I., McKenzie, B. S., Zhou, L., Tadokoro, C. E., Lepelley, A., Lafaille, J. J., et al. (2006), The orphan nuclear receptor RORgammat directs the differentiation program of proinflammatory IL-17+ T helper cells, *Cell*, 126, 6, 1121–1133, doi:10.1016/j.cell.2006.07.035

Jash, A., Sahoo, A., Kim, G. C., Chae, C. S., Hwang, J. S., Kim, J. E., et al. (2012), Nuclear factor of activated T cells 1 (NFAT1)-induced permissive chromatin modification facilitates nuclear factor-B (NF-B)-mediated interleukin-9 (IL-9) transactivation, *Journal of Biological Chemistry*, 287, 19, 15445–15457, doi:10.1074/jbc.M112.340356

Jenks, J. A., Seki, S., Kanai, T., Huang, J., Morgan, A. A., Scalco, R. C., et al. (2013), Differentiating the roles of STAT5B and STAT5A in human CD4+ T cells, *Clinical Immunology*, 148, 2, 227–236, doi:10.1016/j.clim.2013.04.014

Johnston, R. J., Choi, Y. S., Diamond, J. A., Yang, J. A., and Crotty, S. (2012), STAT5 is a potent negative regulator of TFH cell differentiation, *Journal of Experimental Medecine*, 209, 2, 243–250, doi:10.1084/jem.20111174

Kamiya, S., Owaki, T., Morishima, N., Fukai, F., Mizuguchi, J., and Yoshimoto, T. (2004), An indispensable role for STAT1 in IL-27-induced T-bet expression but not proliferation of naive CD4+ T cells, *Journal of Immunology*, 173, 6, 3871–3877, doi:10.4049/jimmunol.173.6.3871

Kanhere, A., Hertweck, A., Bhatia, U., Gokmen, M. R., Perucha, E., Jackson, I., et al. (2012), T-bet and GATA3 orchestrate Th1 and Th2 differentiation through lineage-specific targeting of distal regulatory elements, *Nature Communications*, 3, 1268, doi:10.1038/ncomms2260

Kano, S., Sato, K., Morishita, Y., Vollstedt, S., Kim, S., Bishop, K., et al. (2008), The contribution of transcription factor IRF1 to the interferon-gamma-interleukin 12 signaling axis and TH1 versus TH-17 differentiation of CD4+ T cells, *Nature Immunology*, 9, 1, 34–41, doi:10.1038/ni1538

Kiani, A., Garcia-Cozar, F. J., Habermann, I., Laforsch, S., Aebischer, T., Ehninger, G., et al. (2001), Regulation of interferon-gamma gene expression by nuclear factor of activated T cells, *Blood*, 98, 5, 1480–1488, doi:10.1182/blood.V98.5.1480

Kim, H. P., Imbert, J., and Leonard, W. J. (2006), Both integrated and differential regulation of components of the IL-2/IL-2 receptor system, *Cytokine Growth Factor Reviews*, 17, 5, 349–366, doi:10.1016/j.cytogfr.2006.07.003

Kitamura, N., Kaminuma, O., Mori, A., Hashimoto, T., Kitamura, F., Miyagishi, M., et al. (2005), Correlation between mRNA expression of Th1/Th2 cytokines and their specific transcription factors in human helper T-cell clones, *Immunology and Cell Biology*, 83, 5, 536–541, doi:10.1111/j.1440-1711.2005.01364.x

Kotenko, S. V. and Pestka, S. (2000), Jak-Stat signal transduction pathway through the eyes of cytokine class II receptor complexes, *Oncogene*, 19, 21, 2557–2565, doi:10.1038/sj.onc.1203524

Kurowska-Stolarska, M., Kewin, P., Murphy, G., Russo, R. C., Stolarski, B., Garcia, C. C., et al. (2008), IL-33 induces antigen-specific IL-5+ T cells and promotes allergic-induced airway inflammation independent of IL-4, *Journal of Immunology*, 181, 7, 4780–4790, doi:10.4049/jimmunol.181.7.4780

Laurence, A., Amarnath, S., Mariotti, J., Kim, Y. C., Foley, J., Eckhaus, M., et al. (2012), STAT3 transcription factor promotes instability of nTreg cells and limits generation of iTreg cells during acute murine graft-versus-host disease, *Immunity*, 37, 2, 209–222, doi:10.1016/j.immuni.2012.05.027

Laurence, A., Tato, C. M., Davidson, T. S., Kanno, Y., Chen, Z., Yao, Z., et al. (2007), Interleukin-2 signaling via STAT5 constrains T helper 17 cell generation, *Immunity*, 26, 3, 371–381, doi:10.1016/j.immuni.2007.02.009

Lavenu-Bombled, C., Trainor, C. D., Makeh, I., Romeo, P. H., and Max-Audit, I. (2002), Interleukin-13 gene expression is regulated by GATA-3 in T cells: role of a critical association of a GATA and two GATG motifs, *Journal of Biological Chemistry*, 277, 21, 18313–18321, doi:10.1074/jbc.M110013200

Maitra, U., Davis, S., Reilly, C. M., and Li, L. (2009), Differential regulation of Foxp3 and IL-17 expression in CD4 T helper cells by IRAK-1, *Journal of Immunology*, 182, 9, 5763–5769, doi:10.4049/jimmunol.0900124

Moriggl, R., Topham, D. J., Teglund, S., Sexl, V., McKay, C., Wang, D., et al. (1999), Stat5 is required for IL-2-induced cell cycle progression of peripheral T cells, *Immunity*, 10, 2, 249–259, doi:10.1016/S1074-7613(00)80025-4

Mukasa, R., Balasubramani, A., Lee, Y. K., Whitley, S. K., Weaver, B. T., Shibata, Y., et al. (2010), Epigenetic instability of cytokine and transcription factor gene loci underlies plasticity of the T helper 17 cell lineage, *Immunity*, 32, 5, 616–627, doi:10.1016/j.immuni.2010.04.016

Nakahira, M., Tomura, M., Iwasaki, M., Ahn, H. J., Bian, Y., Hamaoka, T., et al. (2001), An absolute requirement for STAT4 and a role for IFN-gamma as an amplifying factor in IL-12 induction of the functional IL-18 receptor complex, *Journal of Immunology*, 167, 3, 1306–1312, doi:10.4049/jimmunol.167.3.1306

Nakayamada, S., Kanno, Y., Takahashi, H., Jankovic, D., Lu, K. T., Johnson, T. A., et al. (2011), Early Th1 cell differentiation is marked by a Tfh cell-like transition, *Immunity*, 35, 6, 919–931, doi:10.1016/j.immuni.2011.11.012

Nurieva, R. I., Chung, Y., Martinez, G. J., Yang, X. O., Tanaka, S., Matskevitch, T. D., et al. (2009), Bcl6 mediates the development of T follicular helper cells, *Science*, 325, 5943, 1001–1005, doi:10.1126/science.1176676

Nurieva, R. I., Mai, X. M., Forbush, K., Bevan, M. J., and Dong, C. (2003), B7h is required for T cell activation, differentiation, and effector function, *Proceedings of the National Academy of Sciences U.S.A.*, 100, 24, 14163–14168, doi:10.1073/pnas.2335041100

Ouyang, W., Lohning, M., Gao, Z., Assenmacher, M., Ranganath, S., Radbruch, A., et al. (2000), Stat6-independent GATA-3 autoactivation directs IL-4-independent Th2 development and commitment, *Immunity*, 12, 1, 27–37, doi:10.1016/S1074-7613(00)80156-9

Ouyang, W., Ranganath, S. H., Weindel, K., Bhattacharya, D., Murphy, T. L., Sha, W. C., et al. (1998), Inhibition of Th1 development mediated by GATA-3 through an IL-4-independent mechanism, *Immunity*, 9, 5, 745–755, doi:10.1016/S1074-7613(00)80671-8

Pandiyan, P., Yang, X. P., Saravanamuthu, S. S., Zheng, L., Ishihara, S., O'Shea, J. J., et al. (2012), The role of IL-15 in activating STAT5 and fine-tuning IL-17A production in CD4 T lymphocytes, *Journal of Immunology*, 189, 9, 4237–4246, doi:10.4049/jimmunol.1201476

Parham, C., Chirica, M., Timans, J., Vaisberg, E., Travis, M., Cheung, J., et al. (2002), A receptor for the heterodimeric cytokine IL-23 is composed of IL-12Rbeta1 and a novel cytokine receptor subunit, IL-23R, *Journal of Immunology*, 168, 11, 5699–5708, doi:10.4049/jimmunol.168.11.5699

Park, K., Park, J. H., Yang, W. J., Lee, J. J., Song, M. J., and Kim, H. P. (2012), Transcriptional activation of the IL31 gene by NFAT and STAT6, *Journal of Leukocyte Biology*, 91, 2, 245–257, doi:10.1189/jlb.0111020

Paul, W. E. and Zhu, J. (2010), How are T(H)2-type immune responses initiated and amplified?, *Nature Reviews Immunology*, 10, 4, 225–235, doi:10.1038/nri2735

Peng, S. L., Gerth, A. J., Ranger, A. M., and Glimcher, L. H. (2001), NFATc1 and NFATc2 together control both T and B cell activation and differentiation, *Immunity*, 14, 1, 13–20, doi:10.1016/S1074-7613(01)00085-1

Rogge, L., D'Ambrosio, D., Biffi, M., Penna, G., Minetti, L. J., Presky, D. H., et al. (1998), The role of Stat4 in species-specific regulation of Th cell development by type I IFNs, *Journal of Immunology*, 161, 12, 6567–6574

Rutz, S., Noubade, R., Eidenschenk, C., Ota, N., Zeng, W., Zheng, Y., et al. (2011), Transcription factor c-Maf mediates the TGF--dependent suppression of IL-22 production in T(H)17 cells, *Nature Immunology*, 12, 12, 1238–1245, doi:10.1038/ni.2134

Sahoo, A., Lee, C. G., Jash, A., Son, J. S., Kim, G., Kwon, H. K., et al. (2011), Stat6 and c-Jun mediate Th2 cell-specific IL-24 gene expression, *Journal of Immunology*, 186, 7, 4098–4109, doi:10.4049/jimmunol.1002620

Saraiva, M., Christensen, J. R., Veldhoen, M., Murphy, T. L., Murphy, K. M., and O'Garra, A. (2009), Interleukin-10 production by Th1 cells requires interleukin-12-induced STAT4 transcription factor and ERK MAP kinase activation by high antigen dose, *Immunity*, 31, 2, 209–219, doi:10.1016/j.immuni.2009.05.012

Schmitt, N., Morita, R., Bourdery, L., Bentebibel, S. E., Zurawski, S. M., Banchereau, J., et al. (2009), Human dendritic cells induce the differentiation of interleukin-21-producing T follicular helper-like cells through interleukin-12, *Immunity*, 31, 1, 158–169, doi:10.1016/j.immuni.2009.04.016

Shoemaker, J., Saraiva, M., and O'Garra, A. (2006), GATA-3 directly remodels the IL-10 locus independently of IL-4 in CD4+ T cells, *Journal of Immunology*, 176, 6, 3470–3479, doi:10.4049/jimmunol.176.6.3470

Szabo, S. J., Dighe, A. S., Gubler, U., and Murphy, K. M. (1997), Regulation of the interleukin (IL)-12R beta 2 subunit expression in developing T helper 1 (Th1) and Th2 cells, *Journal of Experimental Medecine*, 185, 5, 817–824, doi:10.1084/jem.185.5.817

Tone, Y., Furuuchi, K., Kojima, Y., Tykocinski, M. L., Greene, M. I., and Tone, M. (2008), Smad3 and NFAT cooperate to induce Foxp3 expression through its enhancer, *Nature Immunology*, 9, 2, 194–202, doi:10.1038/ni1549

Usui, T., Nishikomori, R., Kitani, A., and Strober, W. (2003), GATA-3 suppresses Th1 development by downregulation of Stat4 and not through effects on IL-12Rbeta2 chain or T-bet, *Immunity*, 18, 3, 415–428, doi:10.1016/S1074-7613(03)00057-8

Usui, T., Preiss, J. C., Kanno, Y., Yao, Z. J., Bream, J. H., O'Shea, J. J., et al. (2006), T-bet regulates Th1 responses through essential effects on GATA-3 function rather than on IFNG gene acetylation and transcription, *Journal of Experimental Medecine*, 203, 3, 755–766, doi:10.1084/jem.20052165

Veldhoen, M., Uyttenhove, C., van Snick, J., Helmby, H., Westendorf, A., Buer, J., et al. (2008), Transforming growth factor-beta 'reprograms' the differentiation of T helper 2 cells and promotes an interleukin 9-producing subset, *Nature Immunology*, 9, 12, 1341–1346, doi:10.1038/ni.1659

Vignali, D. A., Collison, L. W., and Workman, C. J. (2008), How regulatory T cells work, *Nature Reviews Immunology*, 8, 7, 523–532, doi:10.1038/nri2343

Vigne, S., Palmer, G., Martin, P., Lamacchia, C., Strebel, D., Rodriguez, E., et al. (2012), IL-36 signaling amplifies Th1 responses by enhancing proliferation and Th1 polarization of naive CD4+ T cells, *Blood*, 120, 17, 3478–3487, doi:10.1182/blood-2012-06-439026

Villarino, A. V., Tato, C. M., Stumhofer, J. S., Yao, Z., Cui, Y. K., Hennighausen, L., et al. (2007), Helper T cell IL-2 production is limited by negative feedback and STAT-dependent cytokine signals, *Journal of Experimental Medecine*, 204, 1, 65–71, doi:10.1084/jem.20061198

Volpe, E., Servant, N., Zollinger, R., Bogiatzi, S. I., Hupe, P., Barillot, E., et al. (2008), A critical function for transforming growth factor-beta, interleukin 23 and proinflammatory cytokines in driving and modulating human T(H)-17 responses, *Nature Immunology*, 9, 6, 650–657, doi:10.1038/ni.1613

Wang, Y. H., Angkasekwinai, P., Lu, N., Voo, K. S., Arima, K., Hanabuchi, S., et al. (2007), IL-25 augments type 2 immune responses by enhancing the expansion and functions of TSLP-DC-activated Th2 memory cells, *Journal of Experimental Medecine*, 204, 8, 1837–1847, doi:10.1084/jem.20070406

Weil, R. and Israel, A. (2006), Deciphering the pathway from the TCR to NF-kappaB, *Cell Death and Differentiation*, 13, 5, 826–833, doi:10.1038/sj.cdd.4401856

Xu, J., Yang, Y., Qiu, G., Lal, G., Wu, Z., Levy, D. E., et al. (2009), c-Maf regulates IL-10 expression during Th17 polarization, *Journal of Immunology*, 182, 10, 6226–6236, doi:10.4049/jimmunol.0900123

Yang, X., Letterio, J. J., Lechleider, R. J., Chen, L., Hayman, R., Gu, H., et al. (1999), Targeted disruption of SMAD3 results in impaired mucosal immunity and diminished T cell responsiveness to TGF-beta, *EMBO Journal*, 18, 5, 1280–1291, doi:10.1093/emboj/18.5.1280

Yang, X. O., Panopoulos, A. D., Nurieva, R., Chang, S. H., Wang, D., Watowich, S. S., et al. (2007), STAT3 regulates cytokine-mediated generation of inflammatory helper T cells, *Journal of Biological Chemistry*, 282, 13, 9358–9363, doi:10.1074/jbc.C600321200

Yao, Z., Kanno, Y., Kerenyi, M., Stephens, G., Durant, L., Watford, W. T., et al. (2007), Nonredundant roles for Stat5a/b in directly regulating Foxp3, *Blood*, 109, 10, 4368–4375, doi:10.1182/blood-2006-11-055756

Yu, D., Rao, S., Tsai, L. M., Lee, S. K., He, Y., Sutcliffe, E. L., et al. (2009), The transcriptional repressor Bcl-6 directs T follicular helper cell lineage commitment, *Immunity*, 31, 3, 457–468, doi:10.1016/j.immuni.2009.07.002

Yu, Q., Thieu, V. T., and Kaplan, M. H. (2007), Stat4 limits DNA methyltransferase recruitment and DNA methylation of the IL-18Ralpha gene during Th1 differentiation, *EMBO Journal*, 26, 8, 2052–2060, doi:10.1038/sj.emboj.7601653

Zhang, D. H., Yang, L., and Ray, A. (1998), Differential responsiveness of the IL-5 and IL-4 genes to transcription factor GATA-3, *Journal of Immunology*, 161, 8, 3817–3821

Zheng, Y., Josefowicz, S., Chaudhry, A., Peng, X. P., Forbush, K., and Rudensky, A. Y. (2010), Role of conserved non-coding DNA elements in the Foxp3 gene in regulatory T-cell fate, *Nature*, 463, 7282, 808–812, doi:10.1038/nature08750

Zhou, L., Ivanov, I. I., Spolski, R., Min, R., Shenderov, K., Egawa, T., et al. (2007), IL-6 programs T(H)-17 cell differentiation by promoting sequential engagement of the IL-21 and IL-23 pathways, *Nature Immunology*, 8, 9, 967–974, doi:10.1038/ni1488

Zhou, L., Lopes, J. E., Chong, M. M., Ivanov, I. I., Min, R., Victora, G. D., et al. (2008), TGF-beta-induced Foxp3 inhibits T(H)17 cell differentiation by antagonizing RORgammat function, *Nature*, 453, 7192, 236–240, doi:10.1038/nature06878

Zhu, J., Cote-Sierra, J., Guo, L., and Paul, W. E. (2003), Stat5 activation plays a critical role in Th2 differentiation, *Immunity*, 19, 5, 739–748, doi:10.1016/S1074-7613(03)00292-9

Zhu, J., Min, B., Hu-Li, J., Watson, C. J., Grinberg, A., Wang, Q., et al. (2004), Conditional deletion of Gata3 shows its essential function in T(H)1-T(H)2 responses, *Nature Immunology*, 5, 11, 1157–1165, doi:10.1038/ni1128

Ziegler-Heitbrock, L., Lotzerich, M., Schaefer, A., Werner, T., Frankenberger, M., and Benkhart, E. (2003), IFN-alpha induces the human IL-10 gene by recruiting both IFN regulatory factor 1 and Stat3, *Journal of Immunology*, 171, 1, 285–290, doi:10.4049/jimmunol.171.1.285
